# Gender differences in plasma element concentrations and associations between selenoprotein P and iron metabolism in a community-based cohort study

**DOI:** 10.1038/s41598-025-10581-2

**Published:** 2025-07-13

**Authors:** Yoshiro Saito, Misaki Shimizu, Mitsuharu Sato, An Masuda, Kotoko Arisawa, Keiko Taguchi, Takashi Toyama, Ikuko N. Motoike, Kengo Kinoshita, Seizo Koshiba, Masayuki Yamamoto, Toshinari Takamura

**Affiliations:** 1 Laboratory of Molecular Biology and Metabolism, Graduate School of Pharmaceutical Sciences, Tohoku University, 6-3 Aoba, Aramaki, Aoba-ku, Sendai, Miyagi 980-8578 Japan; 2 Tohoku Medical Megabank Organization, Tohoku University, Sendai, 980-8573 Japan; 3 Department of Endocrinology and Metabolism, Kanazawa University Graduate School of Medical Sciences, Kanazawa, Ishikawa 920-8640 Japan

**Keywords:** Multi-elemental analysis, Essential trace elements, Heavy metals, Selenoprotein P, Tohoku Medical Megabank, Community-based cohort study, Biochemistry, Environmental sciences, Biomarkers

## Abstract

Essential trace elements, such as iron (Fe) and selenium (Se), play physiological roles in our body, whereas environmental toxic metals, such as arsenic (As), cadmium (Cd), and mercury (Hg), are known to be associated with various disease risks. However, the relationship between elements, biochemical parameters, and lifestyle habits based on multi-elemental analysis in healthy individuals has not been fully verified. Multi-elemental analysis is useful for evaluating the change in the concentration of these elements and metals. In the present study using totally 100 µL plasma samples from the Tohoku Medical Megabank (TMM) community-based cohort study (total of 506 specimens), we conducted a multi-elemental analysis to evaluate 14 elements in generally healthy subjects. We further determined Se-transporter selenoprotein P levels using the originally developed ELISA method, since increases and decreases in selenoprotein P levels are associated with various disease risks. Multiple correlation analyses between the obtained measured values and several factors suggest that elements such as Fe, Se, and Hg, as well as selenoprotein P levels, are associated with gender differences. We also found that factors such as Fe, Se, As, Hg, hematocrit value, hemoglobin (Hb) content, and HbA1c are correlated with selenoprotein P levels. Furthermore, correlations between Fe levels and Hb content and between As/Hg and fish consumption were found. These findings demonstrate the suitability of multi-elemental analyses with limited plasma sample amounts, clearly show gender-differentiated elements, and establish a significant relationship between selenoprotein P and Fe metabolism.

Essential trace elements, such as iron (Fe), copper (Cu), zinc (Zn), selenium (Se), and molybdenum (Mo), play physiological roles in the human body and are defined by three states: deficiency, recovery through supplementation, and availability of functional molecules containing the element^1–5^. Each element has characteristic properties and exhibits toxicity depending on the level of exposure. Even in the absence of deficiency or excess, fluctuations in the amount of each essential element can be associated with biological dysfunction and an increased risk of disease^6–8^. On the other hand, industrialization has led to increased environmental concentrations of toxic metals as arsenic (As), cadmium (Cd), mercury (Hg), and other hazardous metals, and exposure to these metals is known to be associated with health hazards and various disease risks^9–11^. To evaluate changes in the amounts of essential elements and hazardous metals, multi-elemental analysis of biological samples using inductively coupled plasma mass spectrometry (ICP-MS) has become increasingly useful as the sensitivity of the instruments improves^12–14^.

Although an essential micronutrient, Se is highly reactive and has potent toxicity; it is known as an element with a particularly narrow recommended range between deficiency and excess^15,16^. The biological function of Se is mainly mediated by selenoproteins, which contain selenocysteine (Sec)—a cysteine analog in which sulfur (S) is replaced with Se—and the reactivity of the element is utilized for redox reactions. Twenty-five types of human selenoproteins have been identified, and they play diverse roles in preserving redox homeostasis in the human body^16,17^. Glutathione peroxidase (GPx), a representative selenoprotein, mediates the reduction of hydroperoxide, and thioredoxin reductase is important for redox regulation. Selenoprotein P (SeP), encoded by the *SELENOP* gene, is a major selenoprotein that is primarily synthesized in the liver and secreted into the plasma^18,19^. SeP is a unique selenoprotein possessing 10 Sec residues and it functions as a Se transporter. Whereas a decrease in the amount of SeP causes a deficiency of selenoproteins and leads to various dysfunctions related to oxidative stress, an excess of SeP induces signal transduction resistance associated with type 2 diabetes. The excess of SeP impairs insulin action in the skeletal muscle and liver and the insulin secretory capacity of pancreatic β cells^20,21^. Thus, both SeP deficiency and excess are harmful, and excess SeP might serve as a useful marker for type 2 diabetes. Previously, our research group originally developed a specific enzyme-linked immunosorbent assay (ELISA) method detecting full-length SeP and total SeP (full-length SeP and its N-terminal fragment) using monoclonal antibodies with known epitopes^22^. However, the regulation of SeP expression remains largely unknown, and the factors influencing SeP levels have yet to be clarified.

In the present study, we aimed to reveal the relationships among elements, SeP expression, biochemical parameters, and lifestyle habits. Plasma samples from 506 healthy individuals in the Tohoku Medical Megabank (TMM) Community-Based Cohort Study were subjected to multi-elemental analyses and SeP measurements. In the TMM project, participants provided information on their lifestyle and other potential health-related factors through a self-reported questionnaire and by supplying blood and urine samples^23,24^. We performed multiple correlation analyses between the obtained values and identified elements associated with gender differences. Additionally, we established correlation factors between SeP levels and these elements.

## Materials and methods

### Chemicals

3,3′,5,5′-Tetramethylbenzidine (TMB) was purchased from Merck (Darmstadt, Germany). Human SeP used as the standard material was purified from human plasma^25^. Briefly, human plasma was mixed with polyethylene glycol, and then proteins in the supernatant were separated using a heparin-Sepharose CL-6B column (Sima-Aldrich, St Louis, MO, USA), Q-Sepharose Fast Flow (Cytiva, Tokyo, Japan), and Ni–NTA-agarose (WAKO, Osaka, Japan). The buffer of the purified SeP was exchanged using a PD-10 gel filtration column (Cytiva), equilibrated with the desired buffer. Human frozen plasma was kindly provided by the Japanese Red Cross Tohoku Block Blood Center (No. 25J0012) and used for the preparation of pooled human plasma. All other chemicals used were of the highest commercially available quality.

### Participants and blood collection

The TMM project, which is conducted by Tohoku University and Iwate Medical University in Japan, was launched to promote creative reconstruction and address medical issues in the aftermath of the Great East Japan Earthquake disaster that occurred on March 11, 2011^23^. In the TMM Community-Based Cohort, which is a population-based adult cohort in Miyagi and Iwate prefectures, more than 80,000 participants were recruited between May 2013 and March 2016. Participants provided information on their lifestyle and other potential health-related factors through a self-reported questionnaire and provided blood and urine samples. The questionnaire items and blood test procedures have been previously described^26^. In addition, specific health-check information was included in this study. The participant flowchart of the TMM Community-Based Cohort Study has been reported^26^. Each participant provided written informed consent for the use of blood samples in scientific research. This study was conducted in accordance with the amended Declaration of Helsinki. Local institutional review boards or independent ethics committees approved the protocol. Additionally, the study was approved by the Institutional Review Board of the Tohoku Medical Megabank Organization (approval number: 2018-4-109).

We collected plasma samples from residents enrolled in the TMM cohort study based on two criteria (Table 1): the baseline survey (criterion #1) and disease information (criterion #2). To compare healthy individuals with the general population, we established two study groups. Group 1 consisted of 261 participants (127 males and 134 females) classified as healthy individuals, with neutral fat (criterion #4), blood pressure (criterion #5), weight (criterion #6), and BMI (criterion #7) all within the normal range at 50–65 years of age (criterion #3). Group 2 consisted of 245 participants (106 males and 139 females) classified as general subjects without any restrictions regarding criteria #4–#7 or age (criterion #3) (Table 1). Initially, applying criteria #4–#7 in Group 1 resulted in a recruitment bias toward participants in their 40 s. To address this issue, the age range was restricted to 50–65 years (criterion #3). Additional details on the inclusion criteria are provided in Table 1.

**Table 1:**
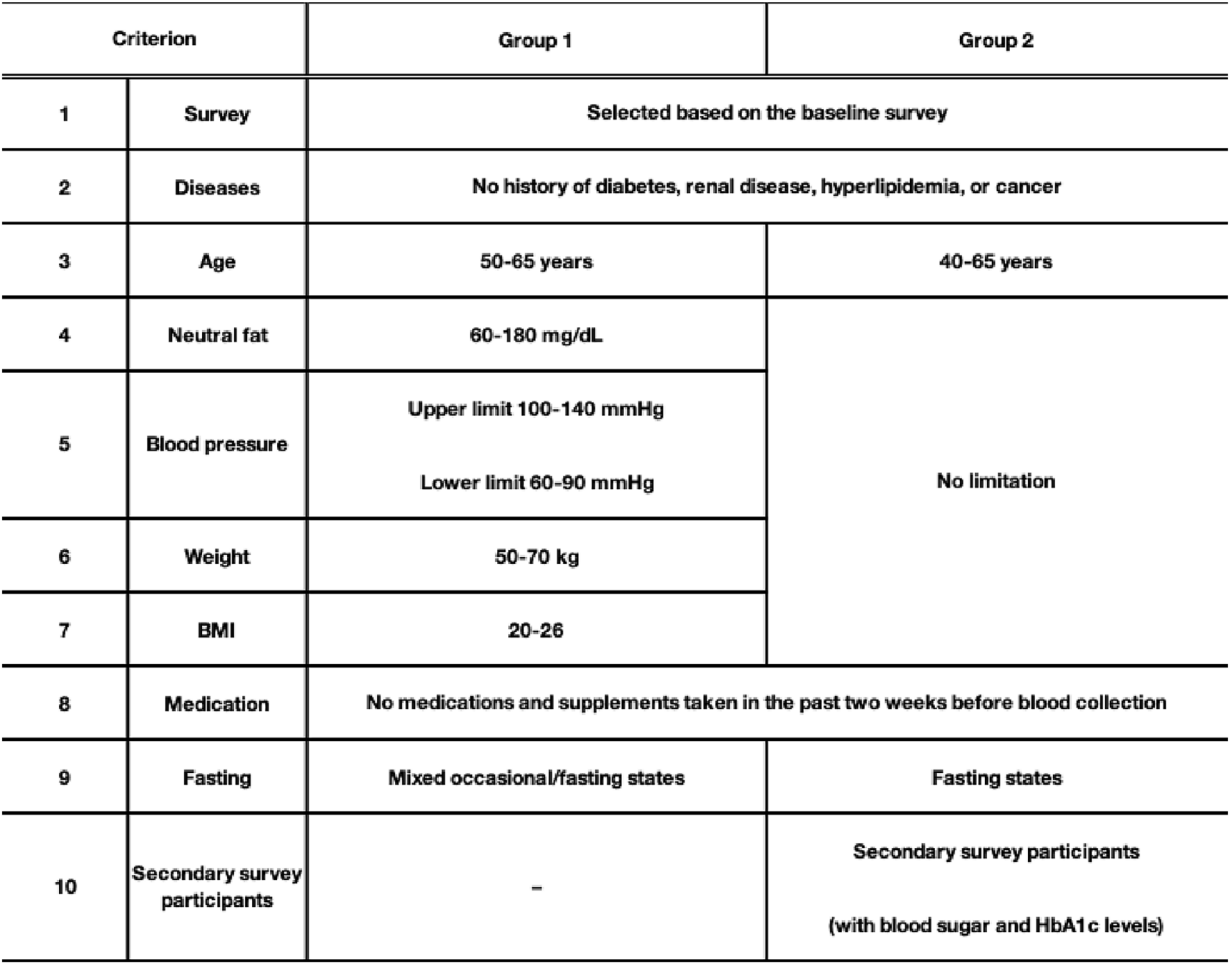
Recruitment criteria for participants in Group 1 and Group 2 from the Tohoku Medical Megabank (TMM) Community-Based Cohort Study. To evaluate the relationship between elemental concentrations, biochemical parameters, and lifestyle habits in both healthy individuals and the general population, plasma samples were randomly selected from residents enrolled in the TMM cohort study. Two groups were defined: Group 1 (n = 261) consisted of healthy individuals who met the normal range criteria for items #4–7, while Group 2 (n = 245) included general participants without these restrictions.

### Determination of elemental concentration by inductively coupled plasma mass spectrometry (ICP-MS)

A total of 506 plasma samples (261 for Group 1 and 245 for Group 2) and reference materials were subjected to multi-elemental analysis by ICP-MS. All samples were stored at – 80 °C until analysis. Human plasma samples (50 μL) were mixed with 0.25 mL of 70% nitric acid and then digested using the microwave sample preparation system ETHOS EASY (Milestone General, Kanagawa, Japan), which is equipped with up to eight high-pressure quartz vessels (rotor-type 8NXQ 80), for 30 min. This system operated with a maximum pressure and temperature of 80 bar and 160 °C, respectively. The pressure and temperature within each vessel were monitored throughout all runs. After digestion and cooling, the resulting solutions were diluted with ultra-pure water to a final volume of 1.0 mL (final concentration: 17.5% nitric acid) for ICP-MS analysis. For the determination of Na, Mg, P, S, K, and Ca, the digested solutions above were further diluted tenfold with 17.5% nitric acid. Elemental levels were measured using an Agilent 8900 ICP-MS fitted with an auto-sampler (Agilent Technologies, Santa Clara, CA, USA). The ICP-MS was operated in collision/reaction cell mode with He and/or O_2_ gas to minimize polyatomic interferences. The internal standard solution, containing Be, Y, In, Te, and Bi, was diluted with 17.5% nitric acid and injected into ICP-MS online with samples. Instrumental settings for ICP/MS analysis and standard elemental solutions for calibration are shown in Supplementary Table 1. The external calibration curve method combined with an internal standard was used for the quantification. The monitored isotopes were as follows: ^9^Be, ^23^Na, ^24^Mg, ^31^PO, ^32^SO, ^39^K, ^44^Ca, ^56^Fe, ^63^Cu, ^66^Zn, ^75^AsO, ^78^SeO, ^89^Y, ^95^Mo, ^111^Cd, ^115^In, ^125^Te, ^201^Hg, and ^209^Bi. A typical standard curve is shown in Supplementary Fig. 1a and 1b. Daily optimization of the ICP-MS instrument was performed according to the manufacturer’s recommendations, using 1 µg/L of Li, In, and U isotopes to evaluate the analytical response.

### Determination of mercury by atomic absorption spectrometry

Atomic absorption spectrometry of mercury using thermal vaporization was performed using an MA-3 (Nippon Instruments Corporation, Osaka, Japan). Briefly, 100 µL of diluted plasma was subjected to a ceramic sample tray and heated to 850 ℃ for 5 min to vaporize the mercury in the sample. The standard mercury solution for calibration was prepared using mercury chloride, and the external calibration curve method was used for quantification.

### Measurement of selenoprotein P

For the measurement of plasma SeP concentrations, a sandwich ELISA system with different capture antibodies was used^27^. In the BD1 method for measuring both the full-length (FL)-SeP and the N-terminal fragment, the cloned BD1 antibody, recognizing the SeP N-terminal region, was used as the capture antibody, and clone AH5, recognizing the N-terminal region of SeP, was conjugated with horseradish peroxidase (HRP) and used as the detection antibody. In the AA3 method for detecting FL-SeP, the clone AA3, recognizing the C-terminal region, and AH5 conjugated with HRP were used for the capture and detection antibodies, respectively. These antibodies were prepared as reported previously^22,27^.

Ninety-six-well microtiter plates were coated for 18 h at 4 °C with 100 μL of rat anti-human-SeP monoclonal antibody BD1 or AA3 (5 μg/mL) in 0.05 M sodium bicarbonate buffer (pH 9.6), which was filtered before use^22,27^. The stock solutions of each antibody (20–30 mg/mL) were stored at − 30 °C. The wells were washed four times with PBS containing 0.05% Tween 20 (200 μL) and incubated at 37 °C with 150 μL of PBS containing Block Ace (UK-B80, KAC, Japan) for 1 h. After washing the wells four times, 50 μL of SeP standard or plasma sample (diluted with PBS, containing 0.05% Tween 20 and 0.1% bovine serum albumin, PBS-Tween-BSA) was added to each well, and incubated at 37 °C for 1 h. After washing the wells four times, 50 μL of HRP-conjugated rat anti-human-SeP monoclonal antibody AH5 (20 μg/mL) was added and incubated at 37 °C for 1 h. Finally, the plates were washed eight times and dried by shaking. TMB peroxidase substrate (50 µL) (5120–0047, SeraCare Life Science, Gaithersburg, MD, USA) was added to each well, and the protein-substrate reaction was allowed to proceed for 10 min in the dark. The reactions were stopped by the addition of 50 μL of 1 M sulfuric acid to each well. Absorbance was read at 450 nm with a SpectraMax iD5 (Molecular Devices, San Jose, CA, USA). At least four measurements were taken for each plasma sample, and the average value was used for analysis. This assay was shown to be highly reproducible in both intra-assay and inter-assay comparisons, with relative standard deviations (RSDs) within 5% for intra-assay and 10% for inter-assay determinations.

### Questionnaire of food intake

We obtained information on dietary intake from the Food Frequency Questionnaire (FFQ), which was collected in the TMM project^28^. Briefly, the FFQ is a self-administered questionnaire developed to investigate dietary habits and nutritional intake by assessing average dietary intake over the past year. These FFQs were used to estimate the habitual intake frequency and portion sizes of 130 food items and were adapted from the FFQ used in the Japan Public Health Center-based prospective (JPHC) study^28–30^. A unique option for intake frequency, "constitutionally unable to consume," was added to account for cases in which participants were unable to eat certain food items due to symptoms. The daily intake of each food item was calculated by multiplying the frequency and the portion sizes. Food items were categorized, and the daily intake of each nutrient was calculated based on the 2010 edition of the Standard Tables of Food Composition in Japan (Fifth Revised and Enlarged Edition).

### Statistical analysis

A partial Spearman’s rank correlation was used to calculate the correlation coefficient using the R package ppcor^31^. Age, body mass index (BMI), and gender were introduced as covariates. A correlation analysis was performed between SeP and element concentrations and various health parameters, including blood biochemical test results, urine test results, lifestyle questionnaire responses, and dietary habits. To adjust for multiple comparisons within each category, *P*-values were corrected using the Benjamini–Hochberg method^32^. Outliers were identified and removed using the Grubbs’ test at a significance threshold of *p* = 0.00001^33^. All statistical calculations were performed in R version 4.1.2^34^.

## Results and discussion

### Validation of multi-elemental analysis

To validate the multi-elemental analysis of plasma samples collected in the TMM Community-Based Cohort Study, we first determined the levels of 14 elements in pooled human plasma. Plasma samples were mixed with nitric acid and then digested using a microwave system. After cooling, the resulting solutions were diluted and subjected to ICP-MS analysis. Intra-day and inter-day variations were examined by conducting four replicate analyses using ICP-MS, and the RSD was found to be less than 10% for intra-day and less than 16% for inter-day sample analysis (Table 2). We also evaluated spiked samples by adding known, equal amounts of each element to human plasma to assess recovery rates. As shown in Table 2, the recovery of each element was within ± 30%, expect for Cd, which exceeded 72%, suggesting spectral interference in plasma samples. The accuracy of the measured values was further assessed using the human plasma standard reference material SERO203105 (lot number: 1801803) (Table 3). The measured values of Cd levels in SERO203105 were reasonable; however, due to the suggested spectral interference, Cd values were excluded from the statistical analysis. All elements were detected using our method, with concentrations falling within a similar range to reported values and within the 95% confidence interval (Table 3). For S, As, and Mo, the 95% confidence interval was not assigned in SERO203105; however, the intra-day and inter-day variations were within 10% and 20%, respectively, and the recovery rates were appropriate (Tables 2 and 3). Considering the 95% confidence intervals of other elements, we determined that these values were reliable. For Hg, its concentrations were near the lowest limit of the 95% confidence interval (Table 3). We further verified the Hg concentration using the atomic absorption spectrometry and found that the difference between the Hg concentration determined by our ICP-MS method (1619 pg/mL) and that measured by the atomic absorption spectrometry (1750 pg/mL) was less than 10% and within the 95% confidence interval (Table 3). The measured Hg values of spiked samples were relatively low, with a recovery rate of only 72%, suggesting that some Hg was lost during sample digestion. The relative standard deviation of the Hg measurements was less than 10% for intra-day and less than 15% for inter-day sample analysis, indicating that Hg loss during the digestion was minimal under our experimental condition. Based on the above results, we performed a multi-elemental analysis of plasma samples from the cohort study.

**Table 2:**
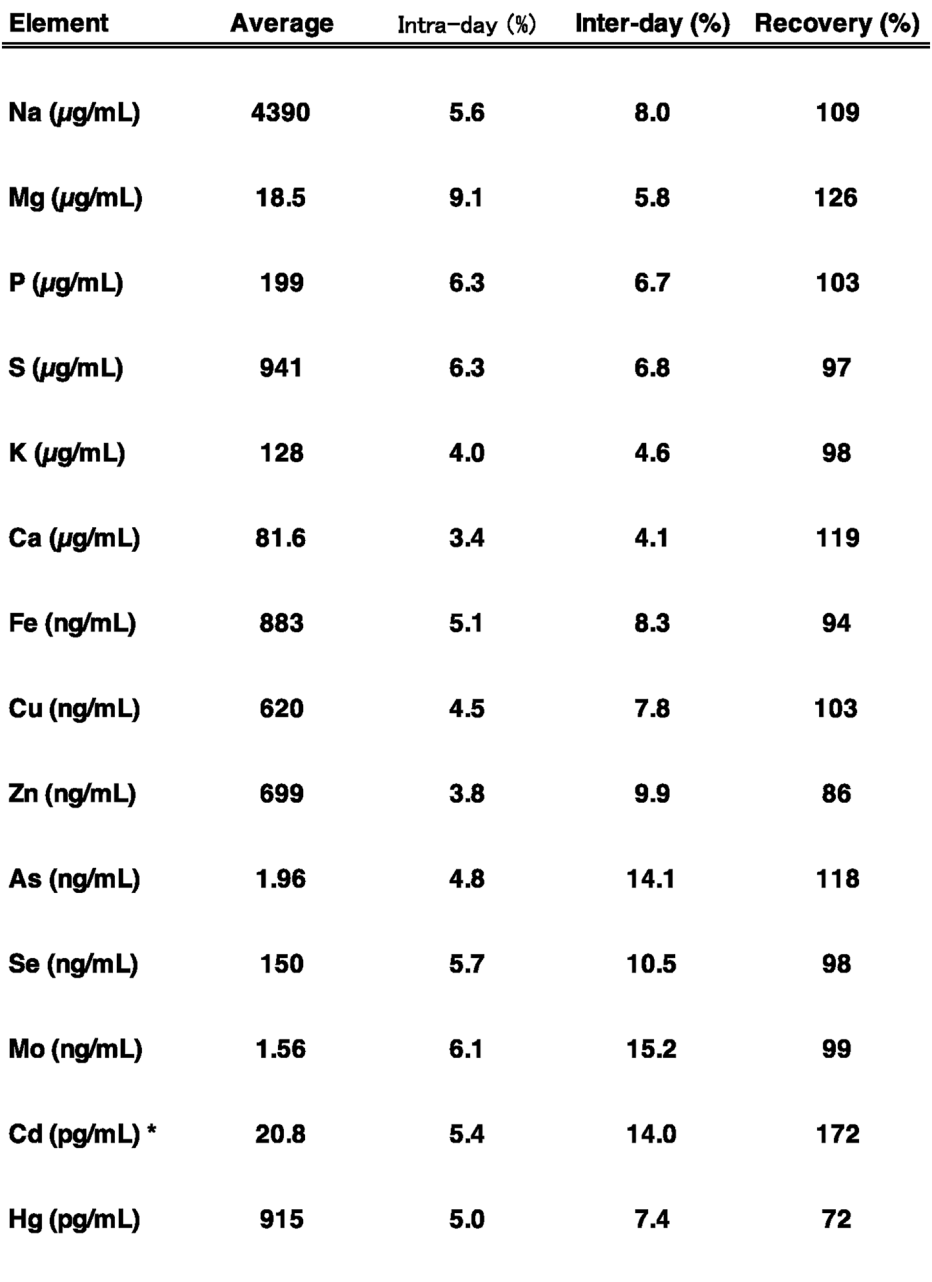
Multi-elemental analysis of pooled human plasma: evaluation of intra-day and inter-day variation and recovery. Plasma samples were mixed with nitric acid and digested using a microwave digestion system. After cooling, the digested solutions were diluted and analyzed by ICP-MS. Intra-day and inter-day variation, as well as recovery, were evaluated. *A spectral interference in the ICP-MS analysis was suggested; therefore, these values were excluded from the statistical analysis.

**Table 3:**
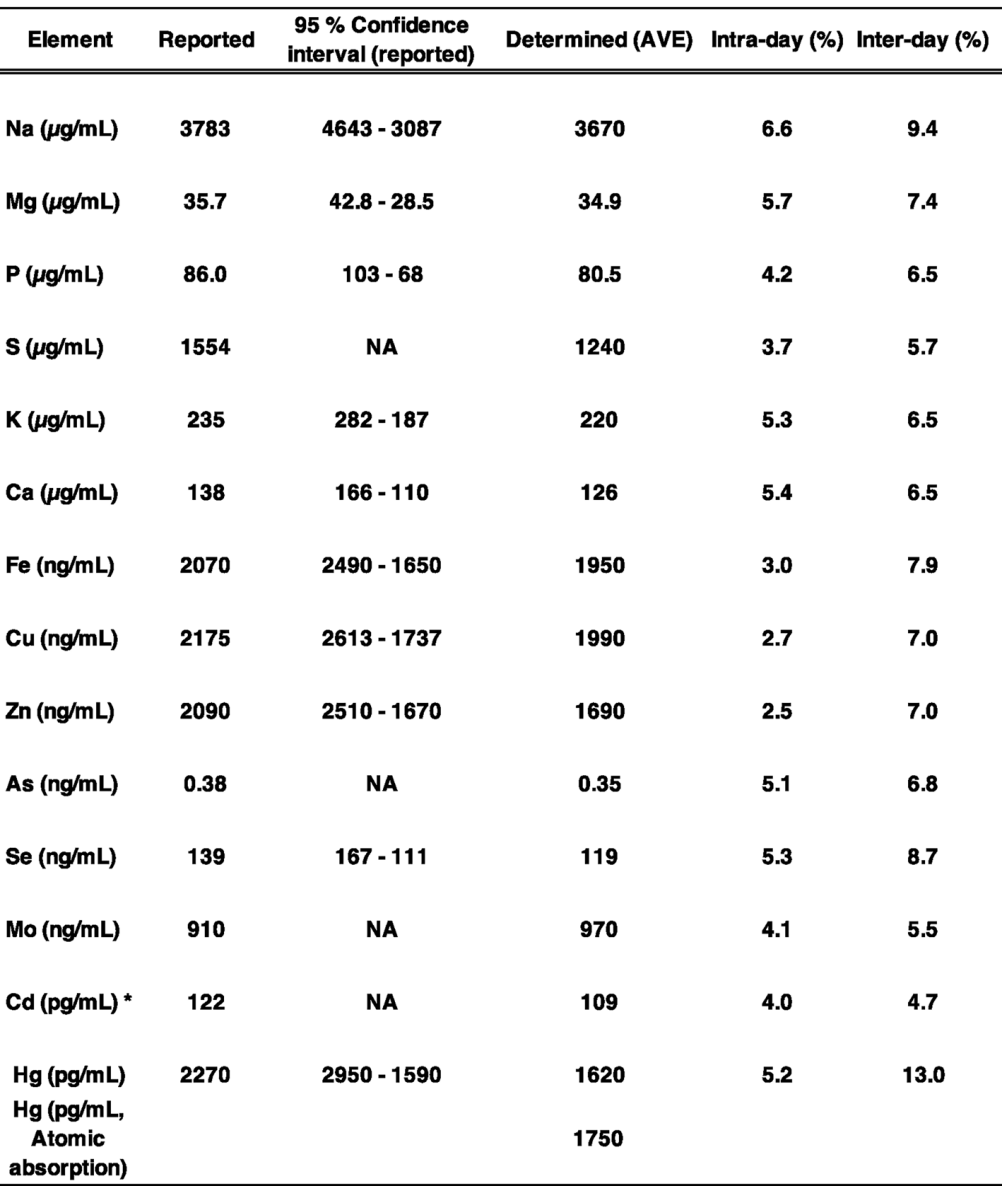
Multi-elemental analysis of human plasma Standard Reference Material (SERO203105) and evaluation of measurement accuracy. The accuracy of the measured values was assessed by analyzing SERO203105. Reported values with their 95% confidence intervals in SERO203105, our measured values, and intra-day and inter-day variations are presentes. *NA* not assigned. *A spectral interference in the ICP-MS analysis was suggested; therefore, these values were excluded from the statistical analysis.

### Multi-elemental analysis of human plasma and gender differences

To evaluate the relationship between elemental concentrations, biochemical parameters, and lifestyle habits in healthy individuals and the general population, we analyzed plasma samples from residents enrolled in the TMM cohort study conducted in Miyagi and Iwate prefectures, Japan^26^. Following recruitment, the TMM cohort study conducted a baseline survey, which included standard biochemical tests, questionnaires, and various omics analyses. The measurement results were stored in the TMM supercomputer. The findings from the baseline survey results are reported previously^26^, and additional information is available on the TMM website (https://www.megabank.tohoku.ac.jp/english/). We collected plasma samples based on two criteria, as described in the Materials and methods section (Table 1). To compare healthy individuals with general population, we established two study groups. Group 1 consisted of individuals classified as healthy, with neutral fat, blood pressure, weight, and BMI all within the normal range, and aged 50–65 years. Group 2 consisted of general subjects without any restrictions regarding the criteria mentioned above or age (Table 1). Plasma samples from all 506 participants were analyzed for SeP concentrations and multi-elemental composition. SeP levels were measured using two assay systems: 1. Total-SeP measurement: Both FL-SeP and its N-terminal fragment were detected using the BD1 antibody, which recognizes the SeP N-terminal region, as the capture antibody. 2. FL-SeP measurement: The AA3 antibody, which recognizes the C-terminal region of SeP, was used as the capture antibody. In both assay systems, the AH5 antibody, which recognizes the N-terminal region, was conjugated with HRP and used as the detection antibody. Table 4 presents the measured concentrations of Total-SeP, FL-SeP, and 14 elements for each group.

**Table 4:**
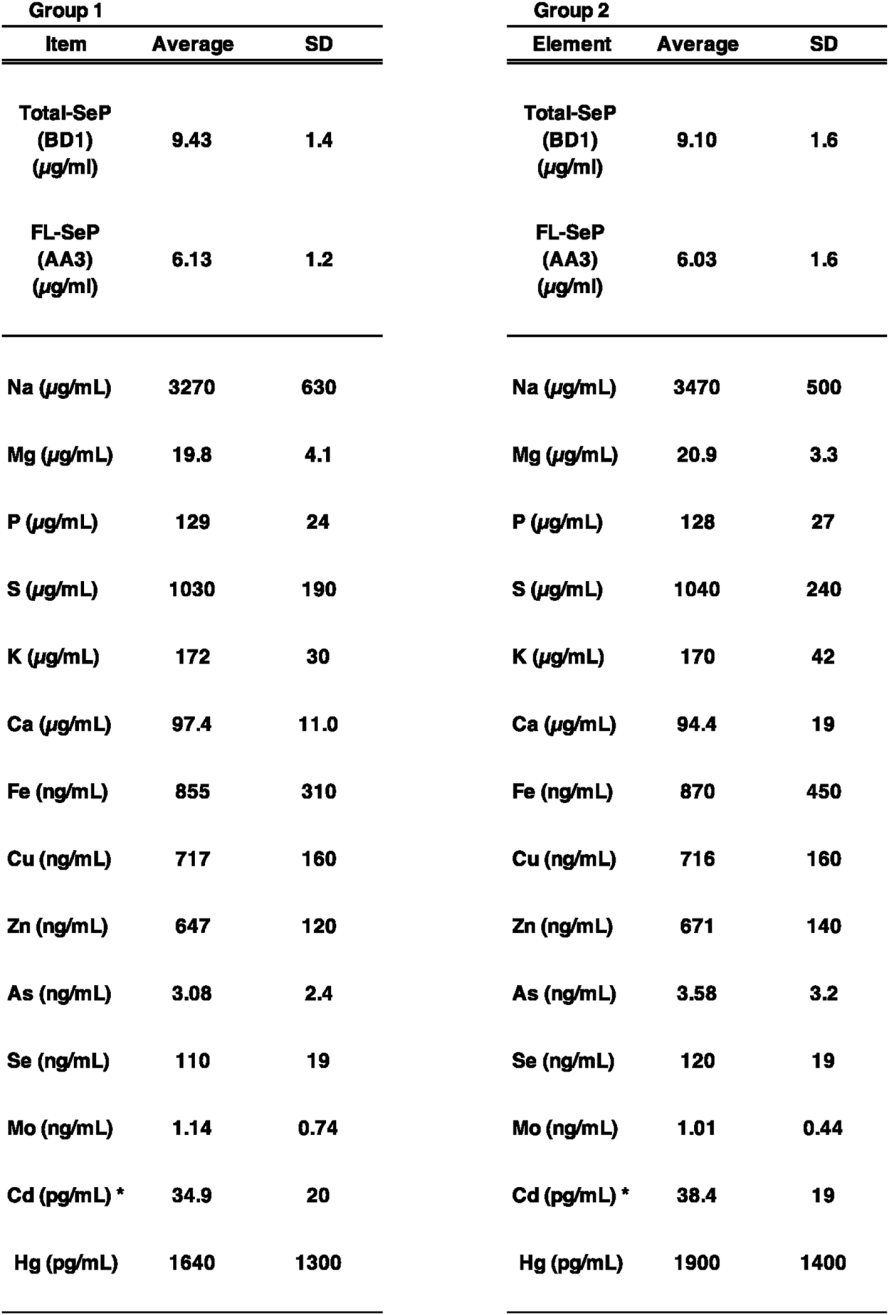
Elemental concentrations and SeP levels in each group. Plasma samples collected from participants in the TMM cohort study were analyzed for both healthy individuals (Group 1) and general subjects (Group 2). The mean and standard deviation (SD) of all measured values, including elemental concentrations and SeP levels, are presented for each group. *A spectral interference in the ICP-MS analysis was suggested; therefore, these values were excluded from the statistical analysis.

We found that both Total-SeP and FL-SeP levels were significantly higher in males than in females in each group (Table 5), which is consistent with previous reports^35–37^. To further investigate gender-related differences, we evaluated each measured parameter in both groups. In Group 1, consisting of healthy individuals, Se levels were significantly higher in males than in females. In contrast, this difference was not significant in Group 2, which represented the general population. The differences of median values in Total-SeP, FL-SeP, and Se levels between males and females in Group 1 were 8.8%, 10.0%, and 5.4% of male values, respectively. In Group 2, Total-SeP and FL-SeP were significantly higher in males, with the differences of median values in Total-SeP, FL-SeP, and Se levels being 8.8%, 9.4%, and 2.5% of male values, respectively. These findings suggest that the gender difference in SeP levels is larger than that in Se levels. For other elements, significant differences in Fe levels (only in Group 2) and Hg levels were observed (Table 5). These results suggest gender-related differences in SeP, essential trace elements, and harmful elements. Previous studies have reported that fish consumption is associated with Hg levels^38,39^. In general, males tend to consume more fish than females^40^. We further analyzed fish intake frequency by gender in the present study and found that males had a higher intake of seafood, bonito, and tuna; however, these differences were not statistically significant (data not shown). The FFQ we used required participants to recall their eating habits over the course of a year, but significant differences have been reported in the results of a one-week dietary record^41,42^. It is considered that a more precise analysis is needed to evaluate these eating habits. Lifestyle differences between male and female participants likely influenced the results of this study. Further research is needed to determine whether these gender differences are primarily due to genetic factors or lifestyle variations.

**Table 5:**
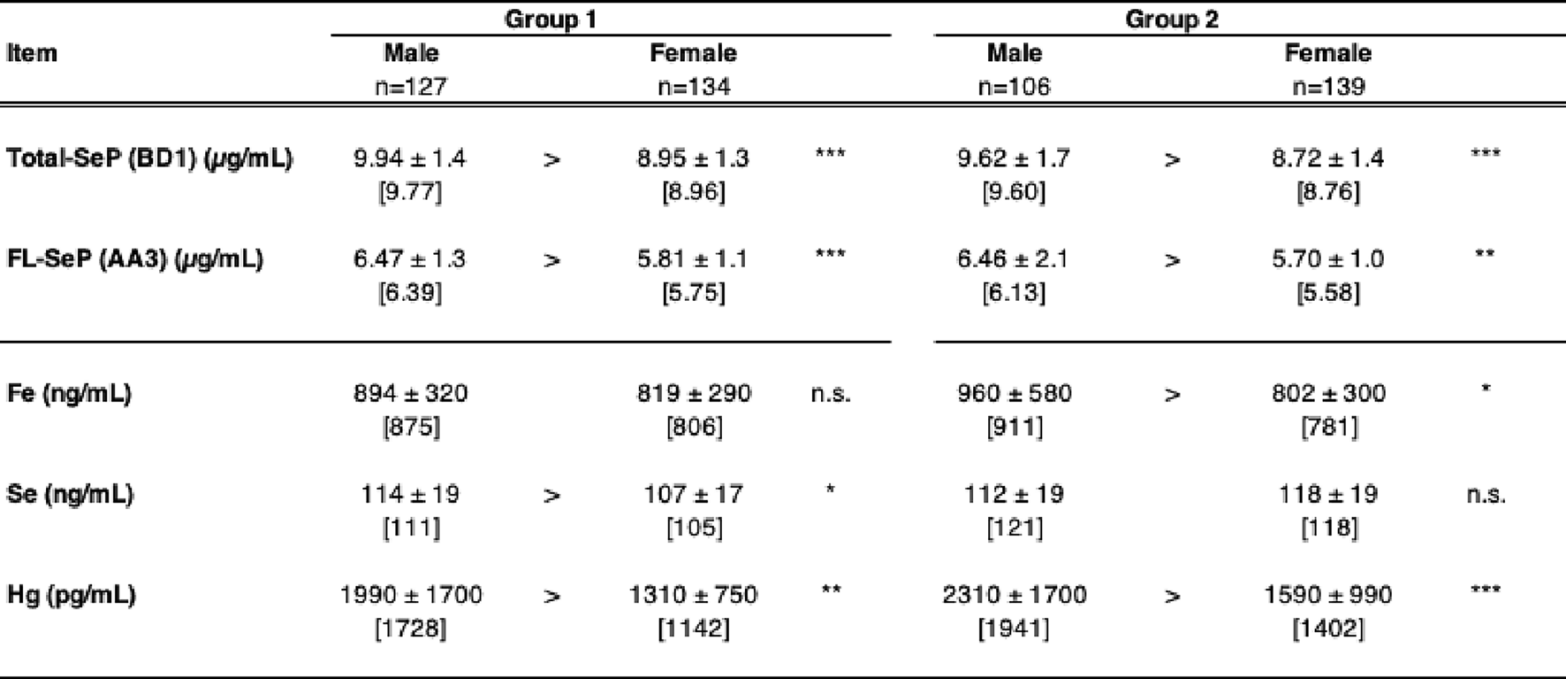
Variables showing significant gender differences in each group. To evaluate the relationships between elemental levels, biochemical parameters, and lifestyle habits in healthy individuals and general subjects, two groups were defined. Gender-specific differences were assessed for each variable within each group. Both Total-SeP and FL-SeP levels were significantly higher in males in both groups. Significant differences in Se, Fe, and Hg concentrations were also observed, as indicated. To evaluate the relationships between elemental levels, biochemical parameters, and lifestyle habits in healthy individuals and general subjects, two groups were defined. Gender-specific differences were assessed for each variable within each group. Both Total-SeP and FL-SeP levels were significantly higher in males in both groups. Significant differences in Se, Fe, and Hg concentrations were also observed, as indicated. The mean and standard deviation are presented for each group, with the median shown in square brackets. *n.s.* not significant. ***p < 0.001, **p < 0.01, *p < 0.05, Wilcoxon test.

Gender-specific differences in SeP levels have been reported in several epidemiological studies^43–45^ and animal experiments^35,36^, suggesting the pre- and post-transcriptional processes may contribute to these differences^35,36^. Previous studies indicate that approximately 53% of human plasma Se is derived from SeP^37^ and that changes in SeP levels may be offset when considering total Se levels. Additionally, the detected Se content of purified SeP was 6.3 Se atoms per SeP molecule^25^, which is lower than the predicted 10 selenocysteine residues per SeP molecule, suggesting a potential link to the observed gender differences in SeP levels. Sexual dimorphism is known to influence Se metabolism and its effects on health^43–45^. Furthermore, Se has been shown to affect sex hormone production, including testosterone synthesis^43–45^. Se plays a direct role in spermatogenesis, and selenoprotein GPX4 in the testis, which is maintained by Se supply via SeP, is essential for spermatozoa structural integrity^46,47^. While SeP expression in males has reproductive significance, its regulation by female hormones remains unclear. Hepatic SeP expression is influenced by multiple factors, including HNF-4α, and is associated with increased levels under conditions of hyperglycemia and excessive alcohol intake^48,49^. Males are generally at a higher risk of developing diabetes than females, which may be related to differences in lifestyle habits^50^. Notably, SeP—but not total Se levels—has been reported to predict future hyperglycemia in the general Japanese population^51^. A positive correlation between SeP levels and diabetes, particularly in males, has been observed^44^. While the relative contributions of genetic and lifestyle factors remain uncertain, lifestyle habits may at least partially contribute to elevated SeP levels in males.

### Multiple correlation analysis between SeP and factors

**Fig. 1:**
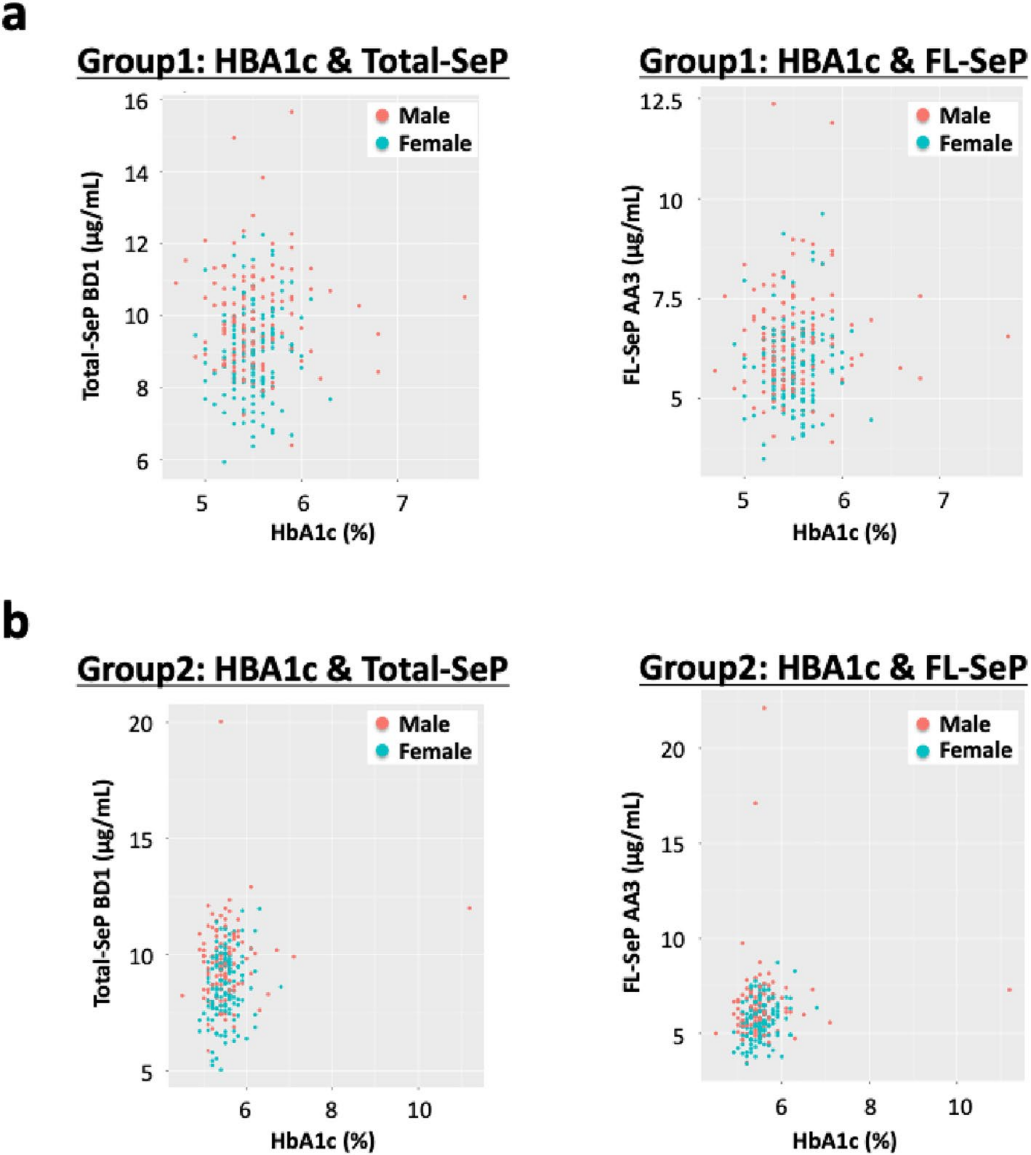
The correlation between total-selenoprotein P (Total-SeP, BD1 method) or full-length selenoprotein P (FL-SeP, AA3 method) and HbA1c in Group 1 (**a**) and Group 2 (**b**). Total-SeP and FL-SeP levels were significantly correlated with HbA1c in Group 2, but no significant correlation was observed in Group 1.

We conducted multiple correlation analyses between SeP levels and various factors, including element concentrations and variables investigated in the TMM Community-Based Cohort such as biochemical test values and questionnaire responses^52^. We conducted multiple correlation analyses between SeP levels and various factors, including element concentrations, biochemical test values, and questionnaire responses. As noted above, SeP and several elemental levels can be influenced by gender, and age and BMI were found to correlate with various biochemical parameters and element concentrations in preliminary analyses. Therefore, we performed partial correlation analyses controlling for age, gender, and BMI, which are considered potential confounders. It is known that male participants tend to show more extreme outliers than females, and these outliers could contribute to observed statistical differences. To mitigate this issue, we used partial Spearman’s rank correlation, a method robust to outliers. In Group 1, we found that Total-SeP and FL-SeP levels were significantly correlated with plasma Se levels but not with HbA1c (Table 6 and Fig. 1a). In contrast, in Group 2, which comprised general subjects not meeting specific metabolic criteria, Total-SeP and FL-SeP levels were significantly correlated with HbA1c levels, suggesting a possible link between SeP and glucose metabolism in this group (Table 6 and Fig. 1b). In Group 2, the variation of SeP levels was high, although the median values were similar to those in Group 1. This is likely due to the lack of metabolic criteria in the recruitment process. Blood glucose levels did not show a significant correlation with SeP levels, potentially due to variability in sample collection timing. Although a linear correlation between SeP and HbA1c was not observed, nonlinear analysis indicated a significant association. The biological effects of Se are known to follow a U-shaped curve, in which both low and high Se levels are associated with increased diabetes risk^26,53^. These findings suggest that SeP levels may be relevant to HbA1c elevation and diabetes susceptibility.

**Table 6:**
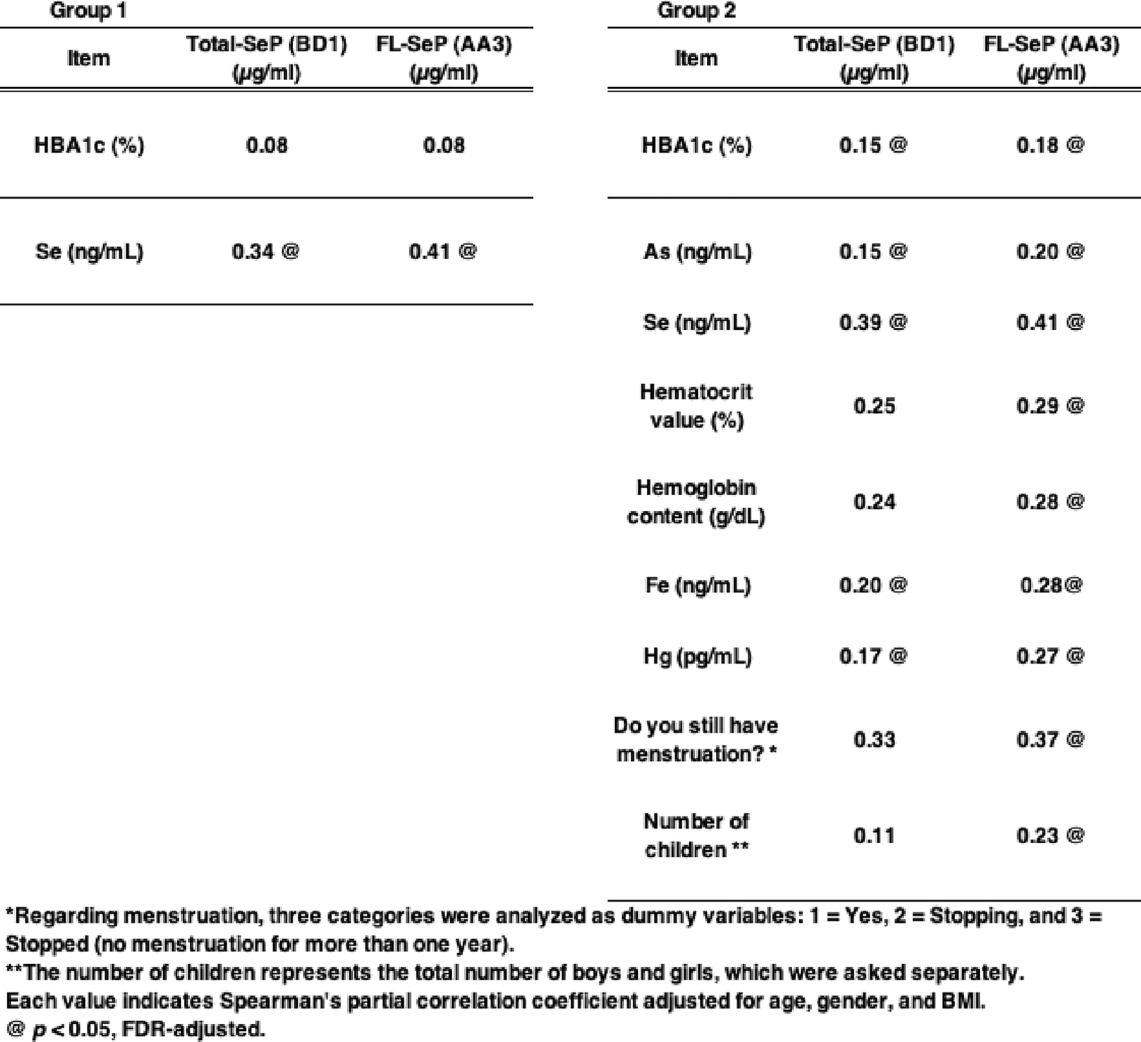
Results of multiple correlation analyses between selenoprotein P (SeP) and various factors. Partial correlation analyses were conducted with age, gender, and BMI included as covariates. Each value indicates the correlation coefficient. Items with statistically significant correlations are highlighted.

**Fig. 2:**
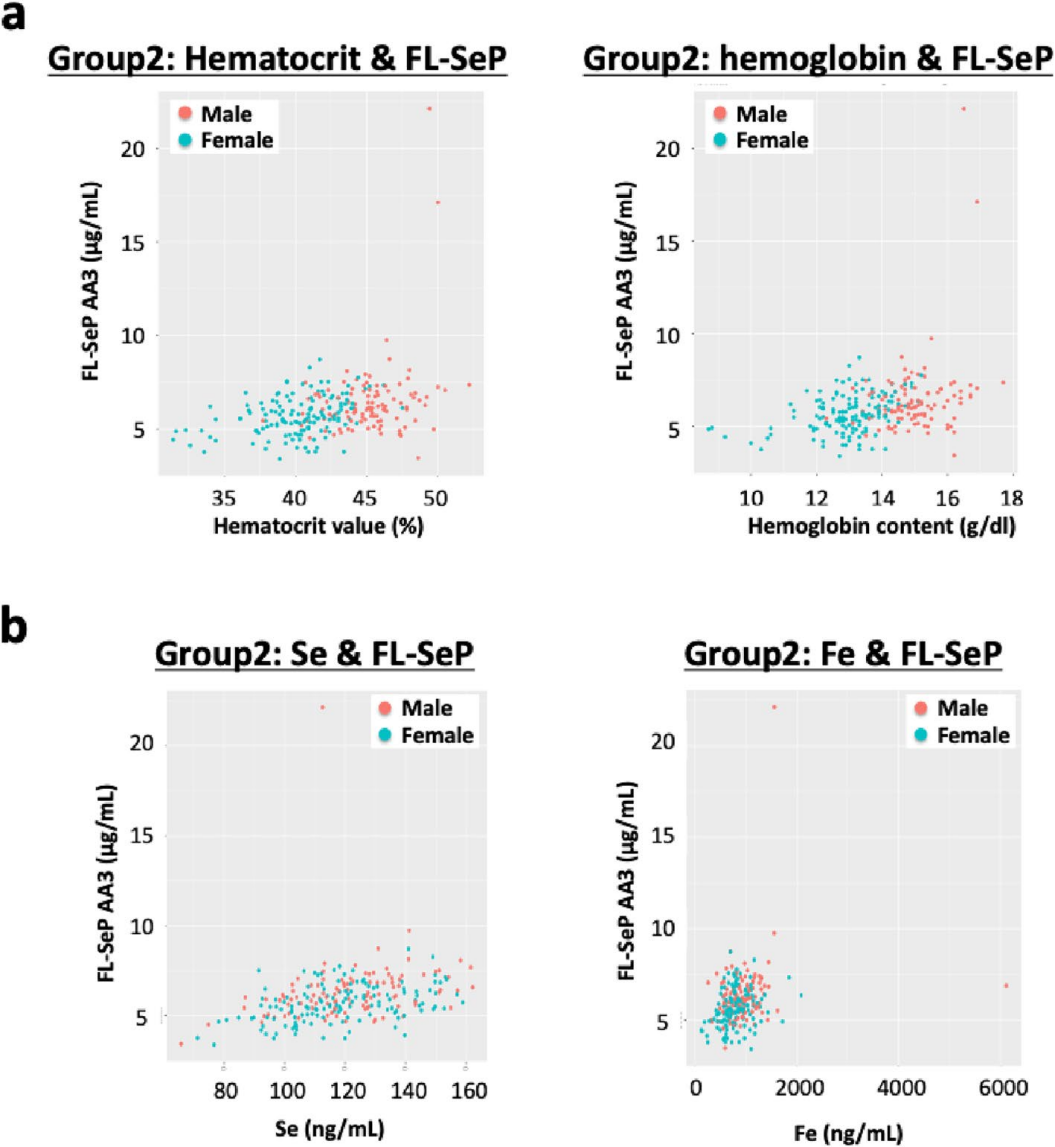
The correlation between full-length selenoprotein P (FL-SeP) and hematocrit values, hemoglobin content (**a**), as well as plasma Se and plasma Fe levels (**b**) in Group 2. FL-SeP levels were significantly correlated with hematocrit values, hemoglobin content, Se, and Fe concentrations in Group 2.

Total-SeP and FL-SeP levels were significantly correlated with several plasma element concentrations, including As, Se, Fe, and Hg in Group 2 (Table 6). In addition to Fe levels, FL-SeP levels were significantly correlated with hematocrit values and Hb content (Table 6), suggesting a link between SeP and Fe metabolism. Correlation analysis with questionnaire items revealed that FL-SeP levels significantly associated with menstruation and number of children (Table 6). Figure 2 shows scatter plots illustrating the positive correlations between FL-SeP levels and hematocrit values, Hb content, Se, and Fe, stratified by sex (Fig. 2). To further evaluate the effects of outliers, we reanalyzed all data after removing outliers identified from continuous variables using Grubbs’ test. Statistical significance was confirmed, and the results are shown in Supplementary Figs. 2 and 3. Taken together, these findings suggest that SeP levels are associated with multiple aspects of metabolism, including not only Se but also Fe, As, and Hg pathways.

The molecular link between SeP and Fe metabolism remains limited; however, recent studies, including a meta-analysis, suggest an association between SeP levels, hemoglobin content, and heart failure^54,55^. Notably, SeP levels in females were correlated with menstruation (Table 6). Previous research has indicated a potential relationship between serum Se deficiency and anemia in specific populations, as well as an association between low Se concentrations and anemia in individuals with heart failure^56,57^. A possible molecular mechanism linking Se and red blood cells has been reported, in which lipid peroxidation is suppressed and the integrity of red blood cell plasma membranes is maintained—a process also associated with vitamin E levels^58,59^. In addition, selenoprotein iodothyronine deiodinase is involved in thyroid hormone metabolism, which is known to play a role in erythropoiesis^60^.

### Multiple correlation analysis between elemental concentrations and other factors

**Fig. 3:**
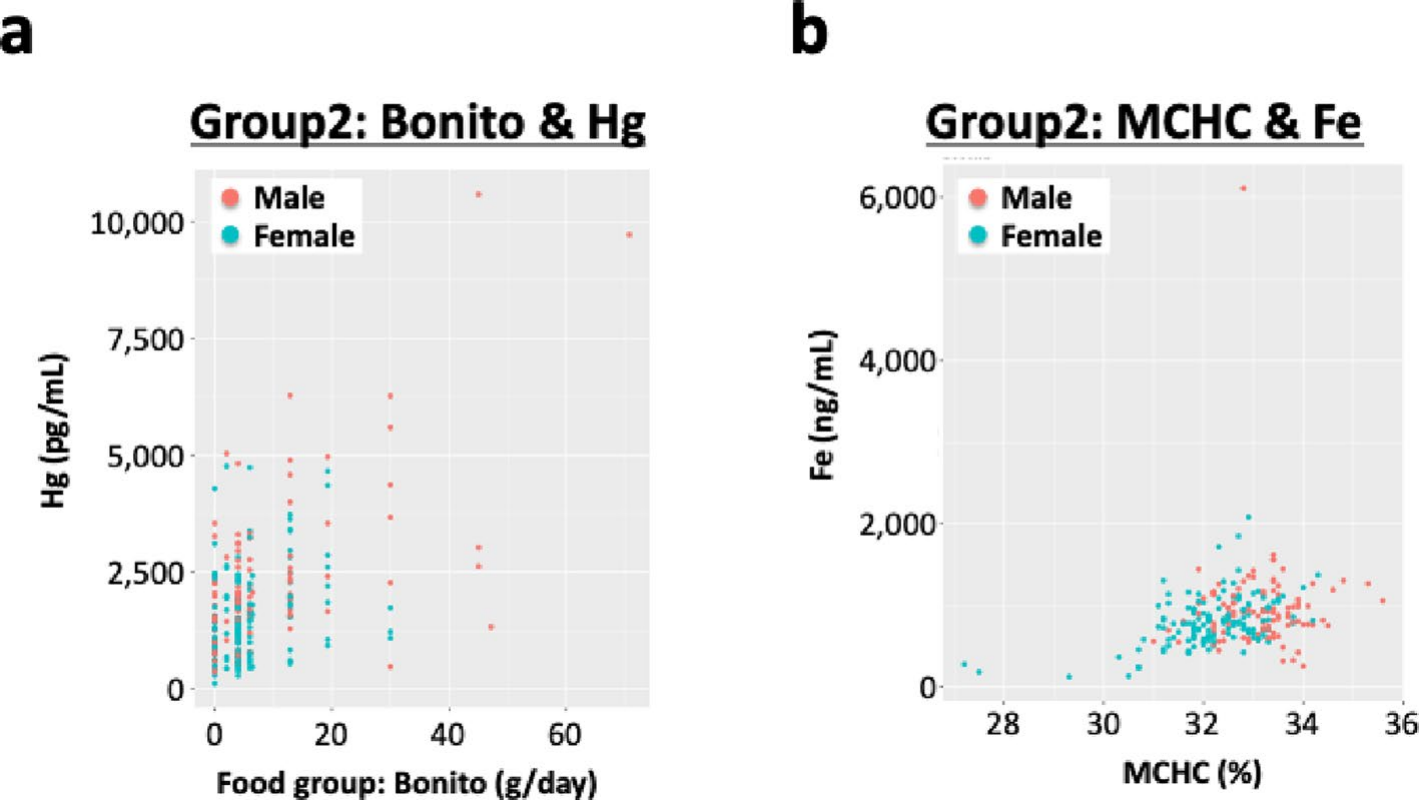
Scatter plots showing the correlation between bonito consumption and plasma Hg levels (**a**), and between mean corpuscular hemoglobin concentration (MCHC) and plasma Fe levels (**b**) in Group 2. Positive correlations were observed between bonito consumption and plasma Hg levels (**a**), and between MCHC and plasma Fe levels (**b**).

We conducted multiple correlation analyses between elemental concentrations and various factors derived from blood biochemical test results and questionnaire responses. The Food Frequency Questionnaire (FFQ) used in the TMM study categorized food groups according to the Standard Tables of Food Composition in Japan (Fifth Revised and Enlarged Edition). Consumption of each food group was estimated based on reported intake frequency and portion size. We found that several elements and heavy metals were significantly correlated with biochemical parameters and dietary/lifestyle questionnaire items (Table 7). In both Groups 1 and 2, positive correlations were observed between plasma Fe levels and Hb content, as well as mean corpuscular Hb concentration (MCHC). Arsenic levels were significantly correlated with seafood consumption (Table 7). Furthermore, Hg levels were also correlated with the frequency of consumption of specific seafoods, including grilled fish, bonito, and tuna, in both groups, suggesting an exposure to As and Hg through dietary intake. In Fig. 3, values were plotted for bonito consumption vs. Hg levels (Fig. 3a) and for MCHC vs. Fe levels (Fig. 3b). We also examined the effects of outliers. After removing outliers based on Grubbs’ test, the statistical significance of the correlations was confirmed (Supplementary Fig. 4). These findings reveal a clear relationship between fish consumption and exposure to harmful elements, as well as multiple associations involving Fe metabolism in red blood cells.

**Table 7:**
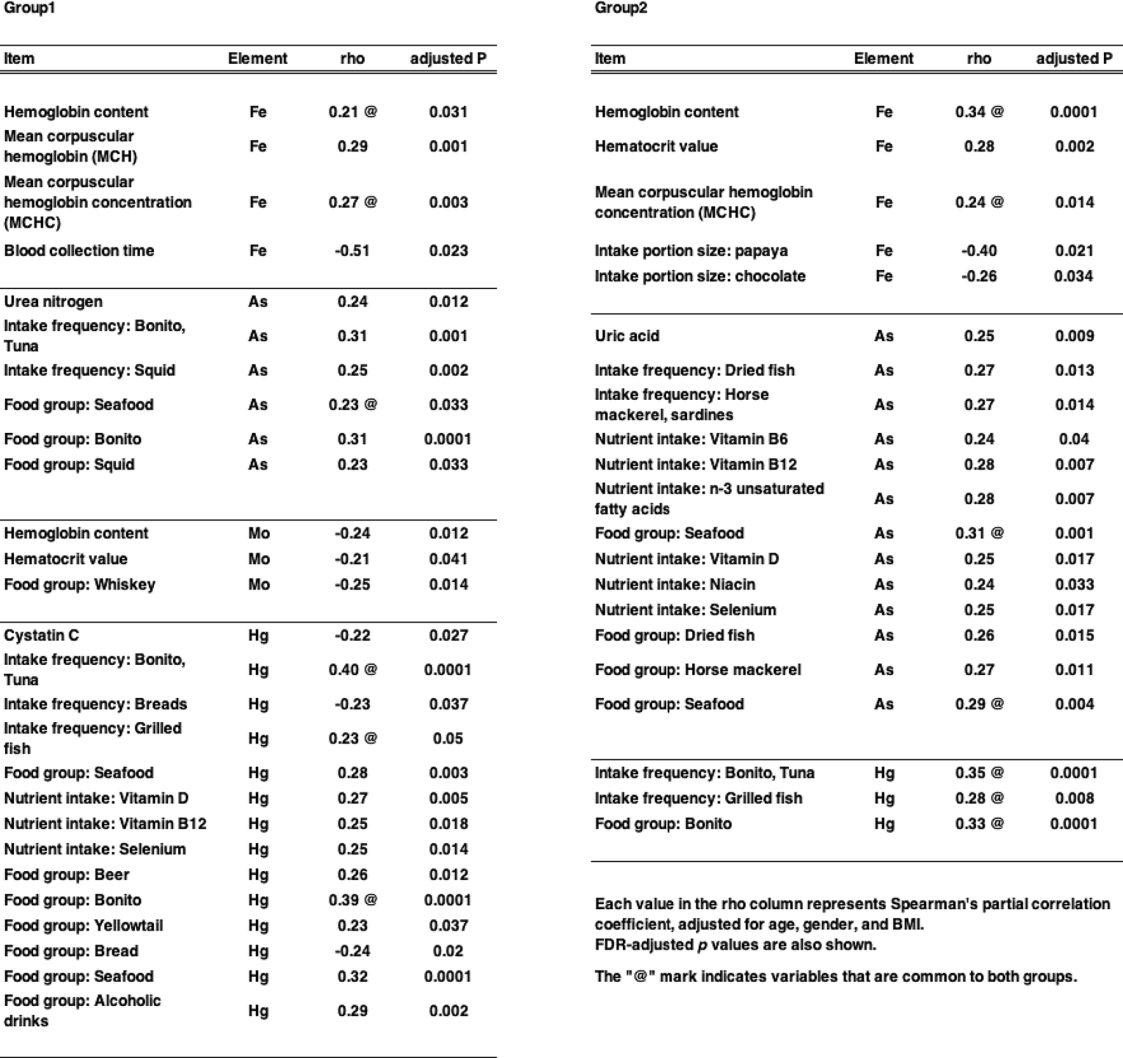
Results of multiple correlation analyses between elemental concentrations and other factors in each group. Correlation analyses were conducted between elemental concentrations and various biological or lifestyle-related factors. Items with statistically significant correlations are highlighted.

As shown in Table 6, As and Hg were significantly correlated with SeP levels in Group 2. These elements are known to bind to SeP and Se, depending on their chemical forms^61,62^. Inorganic As, such as arsenite, can bind to Se and form As-Se complexes. However, in Japan, As is primarily ingested as organic As compounds, such as arsenobetaine, mainly through seafood consumption. Multiple correlation analysis in this study revealed a association between As levels and seafood intake (Table 7)^63^. Organic As compounds are generally less toxic and inert, and the major chemical form of As in blood is organic. Therefore, the significant positive correlation observed between SeP and As levels may not be due to direct complex formation. Interestingly, the questionnaire item “Nutrient intake: Selenium” was also significantly correlated with As levels in Group 2 (Table 7). These findings suggest a possible interaction between Se intake and As levels, as both elements are commonly found in seafood. It is therefore considered that the intake of both Se and As may contribute to the observed positive correlation between SeP and As levels, which warrants further investigation in future studies. In the case of Hg, methylmercury (MeHg) is the predominant chemical form absorbed into the human body through seafood consumption. In the present study, a significant positive correlation was observed between Hg levels and seafood intake (Table 7). Unlike As, MeHg is thought to bind directly to both Se and SeP. It has been reported that approximately 70% of plasma MeHg is bound to SeP^64^. Additionally, co-intake of Se and MeHg has been reported to increase the levels of both elements in several tissues^65^. Collectively, these findings suggest that the simultaneous intake of Se and MeHg contributes to the positive correlation observed between SeP and Hg levels.

We further performed correlation analysis for each gender for the items with previously observed correlations. Items with correlations found in both genders are shown in Supplementary Table 2. While correlations were observed for many items in both genders, some items exhibited larger *p*-values in either gender, suggesting that the correlation patterns may differ between male and female for these items. Although further verification with a larger sample size is necessary, the relationship between Hg and alcohol consumption in Group 1 (with a high *p*-value in female) and the relationship between As and fatty acids/vitamins in Group 2 (with a high *p*-value in male) support this observation.

## Conclusion

The essential role and adverse effects of trace elements and heavy metals have been extensively studied, and many epidemiological studies have investigated individual elements^66–70^. However, epidemiological research utilizing multi-elemental analysis remains limited. In the present study, we applied multi-elemental analysis and SeP measurements to plasma samples from the TMM Community-Based Cohort Study and identified multiple correlations—such as those between SeP and Hg with gender differences, and between SeP and factors including HbA1c, As, Se, Fe, Hg, hematocrit values, and hemoglobin (Hb) content. We also observed associations between plasma trace element concentrations and factors such as biochemical test results and questionnaire responses. These findings provide a valuable foundation for future prospective cohort studies focused on disease risk assessment. Importantly, these results were obtained from only 100 µL of plasma, demonstrating the sensitivity and feasibility of our analytical method. By increasing the number of samples, a Genome-Wide Association Study (GWAS) will be helpful for investigating the genetic backgrounds that influence elemental concentrations, which we plan to pursue as a future project. As this is a prospective cohort study, we will further analyze longitudinal changes in the measured values obtained and assess their relationship to ongoing and future disease risk.

## Supplementary Information (Please change this file to attached file)

Supplementary Information.

## Data Availability

All data needed to evaluate the conclusions are present in the paper. Relevant data are available from the authors upon reasonable request. The requests should be addressed to Yoshiro Saito.
