# Supplementary material for: Gender differences in plasma element concentrations and associations between selenoprotein P and iron metabolism in a community-based cohort study
Source: Sci Rep. 2025 Jul 13;15:25319. doi: 10.1038/s41598-025-10581-2 (PMC12256612; doi:10.1038/s41598-025-10581-2)
Supplement: Supplementary file 1 — Supplementary Information. [file 41598_2025_10581_MOESM1_ESM.pdf]

a STD curve: Na, Mg, P, S, K, Ca and ISTD

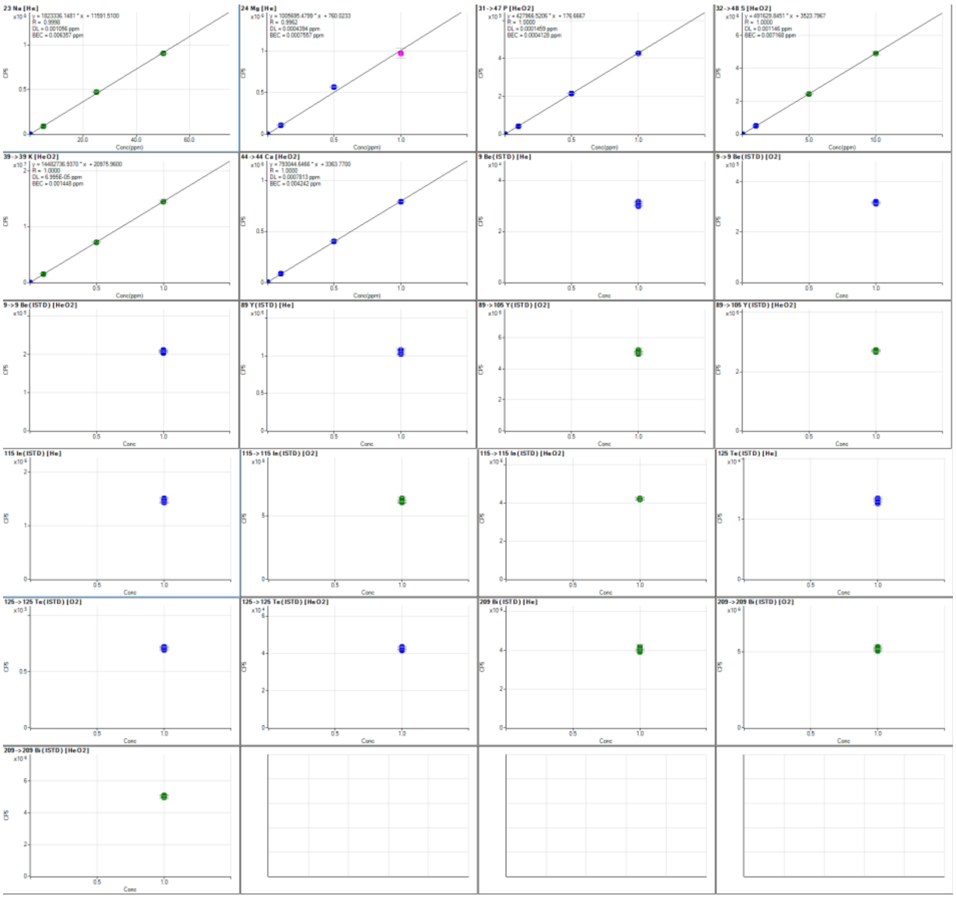

Supplementary Figure 1a Y Saito et al.

b STD curve: Fe, Co, Ni, Cu, Zn, As, Se, Mo, Cd, Hg, and ISTD

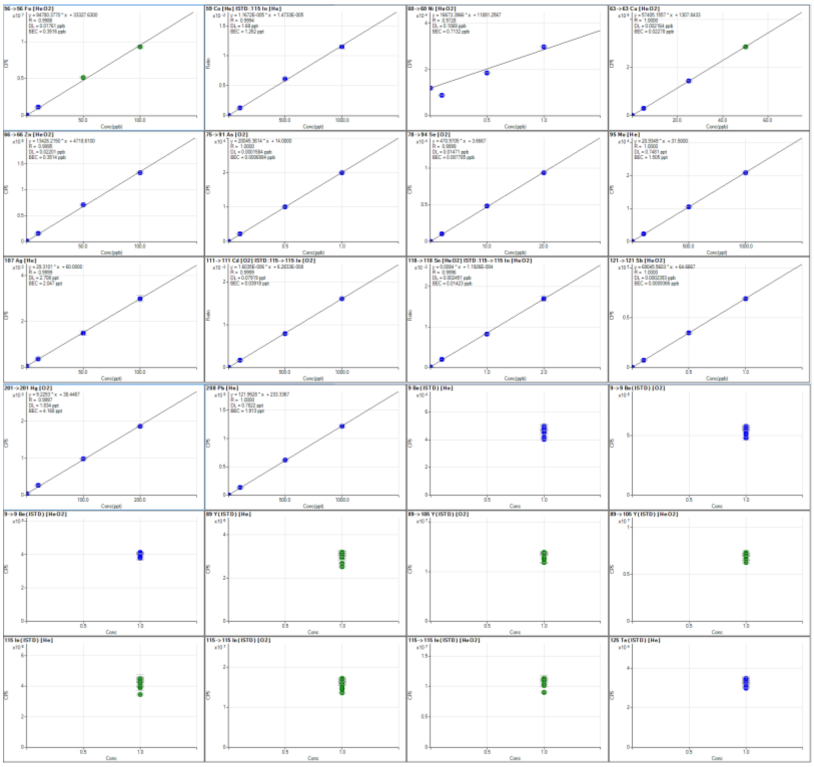

Supplementary Figure 1b Y Saito et al.

Supplementary Figure 1 | Representative standard curves for each element and corresponding internal standard values. The plots show the relationship between counts per second (CPS) and the concentration of each element.

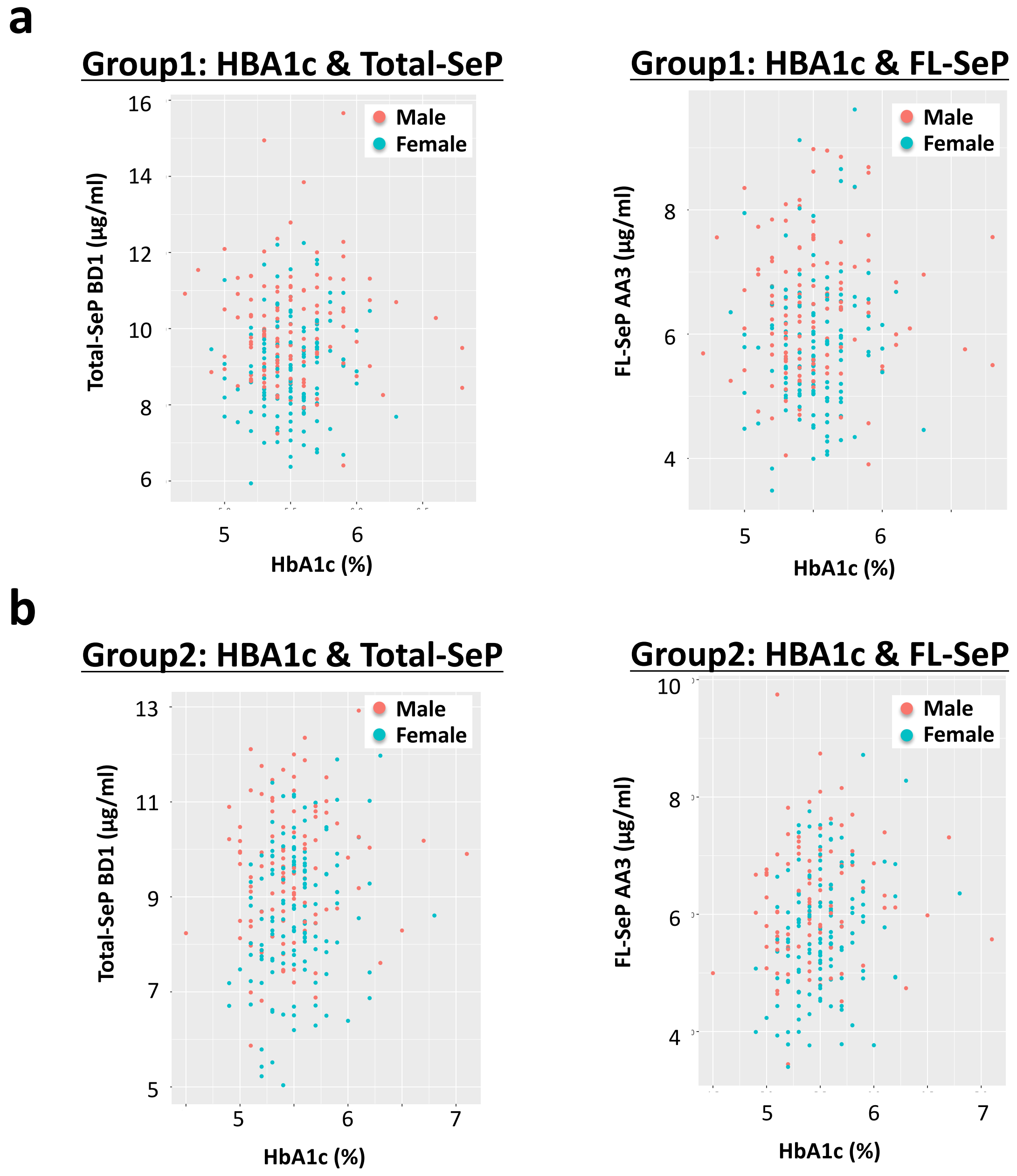

Supplementary Figure 2. Y Saito et al

Supplementary Figure 2 | Correlation between total selenoprotein P (Total-SeP, BD1 method) or full-length selenoprotein P (FL-SeP, AA3 method) and HbA1c in Group 1 (**a**) and Group 2 (**b**), with outliers removed. Outliers were identified and excluded using Grubbs' test. Significant correlations between Total-SeP or FL-SeP levels and HbA1c were observed in Group 2.

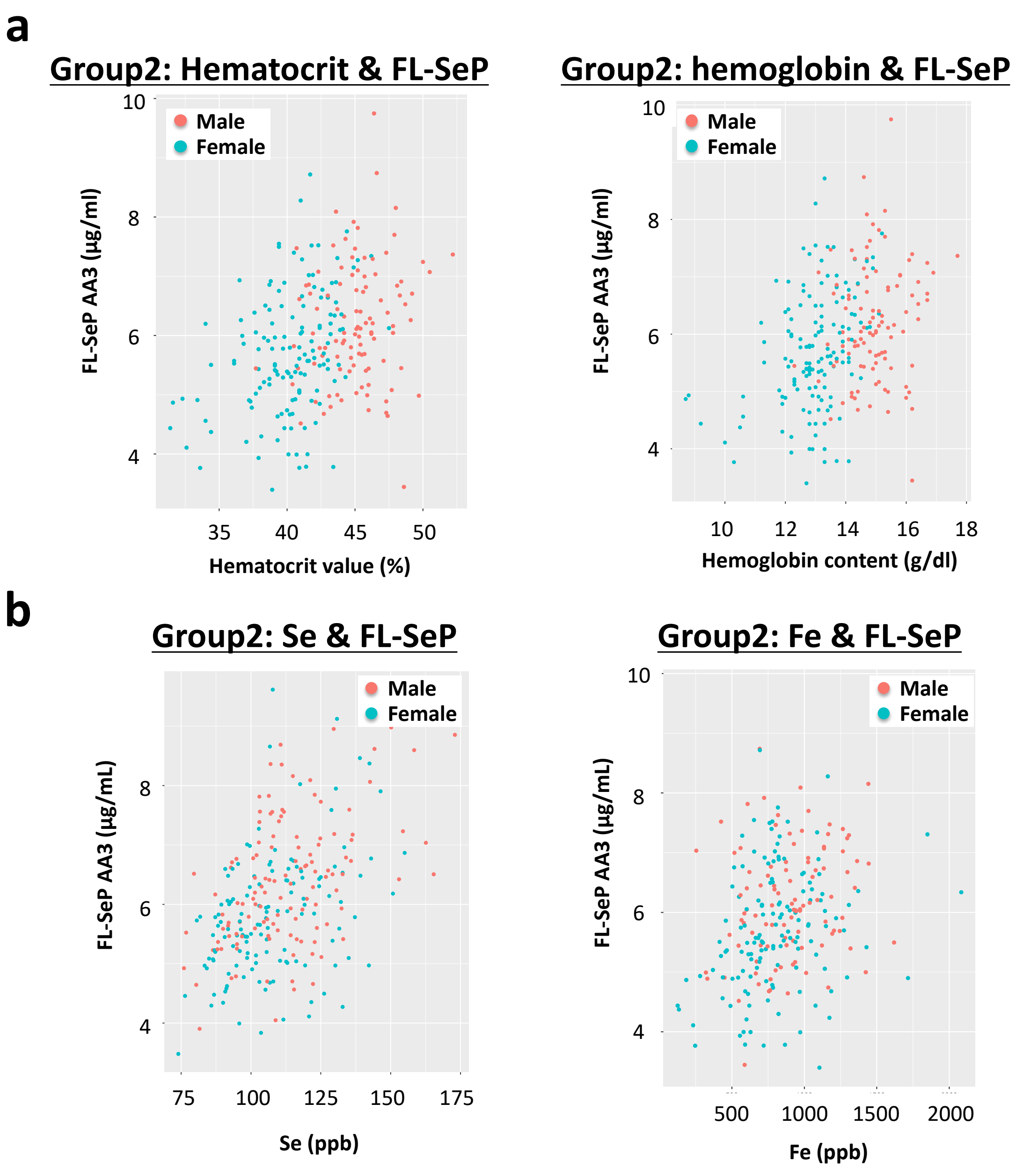

Supplementary Figure 3. Y Saito et al

Supplementary Figure 3 | Correlation between full-length selenoprotein P (FL-SeP) and hematocrit values and hemoglobin content (**a**), or plasma Se and Fe levels (**b**) in Group 2, with outliers removed. Outliers were identified and excluded using Grubbs' test. In Group 2, FL-SeP levels were significantly correlated with hematocrit values, hemoglobin content, Se, and Fe.

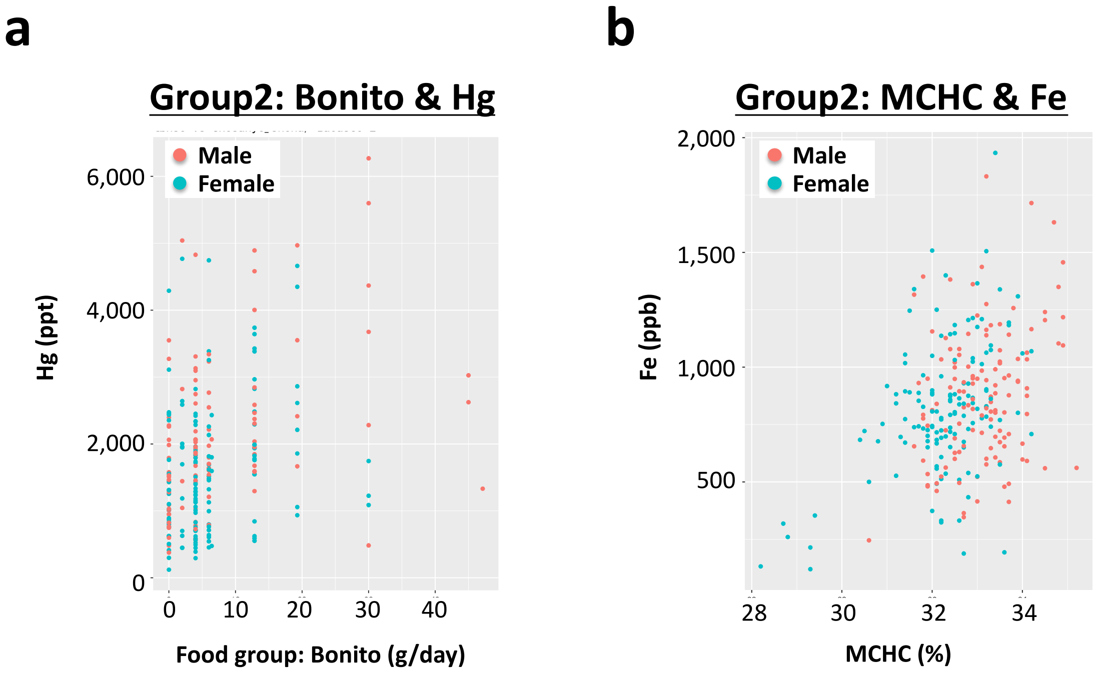

Supplementary Figure 4. Y Saito et al.

Supplementary Figure 4 | Correlation between bonito consumption and plasma Hg levels (**a**), and between mean corpuscular hemoglobin concentration (MCHC) and plasma Fe levels (**b**) in Group 2, with outliers removed. Outliers were identified and excluded using Grubbs' test. Positive correlations were observed between bonito consumption and plasma Hg levels (**a**), and between MCHC and plasma Fe levels (**b**).

Supplementary Table 1. Instrument settings for ICP-MS and standard elemental solutions for calibration. This table summarizes the instrument settings for the ICP-MS, including the collision/reaction cell conditions, and the standard elemental solutions used for calibration in the present study.

Item	Instrument setting for Agilent 8900 ICP-MS	
RF power	1550	watts
Plasma gas flow	15.0	L/min
Auxiliary Gas	0.9	L/min
Neblizer gas flow	1.03	L/min
Nebulizer type	Micromist	
Peristaltic pump speed	0.1	rps
Spray chamber temp	2	°C
Sampler/skimmer cones	Nickel	
Ion lenses model	x-Lens	
Scanning mode	Peak hopping	
Peak pattern	1	point
Replicates	3	
Sweeps/ replicate	10	

Item	Instrument setting for collision/reaction cell	
He mode		
He flow rate	12	mL/min
O2 mode		
O2 flow rate	30	%
HeO2 mode		
He flow rate	12	mL/min
O2 flow rate	10	%

Catalog Number of standard elemental solution (Company Name)	Measured elements
#099-07311 (FUJIFILM)	In
#207-20771 (FUJIFILM)	Te
#168-28331 (FUJIFILM)	P
#198-18601 (FUJIFILM)	S
#096-07321 (FUJIFILM)	Fe
#036-25341 (FUJIFILM)	Cu
#260-02241 (FUJIFILM)	Zn
#47012-1B (KANTO CHEMICAL)	Y
#04881-1B (KANTO CHEMICAL)	Bi
#04869-1B (KANTO CHEMICAL)	Be
#25840-2B (KANTO CHEMICAL)	Mg
#32832-2B (KANTO CHEMICAL)	K
#07998-2B (KANTO CHEMICAL)	Ca
#01805-1B (KANTO CHEMICAL)	As
#37808-1B (KANTO CHEMICAL)	Se
#25828-1B (KANTO CHEMICAL)	Hg
#5190-8454 (Ajilent)	Na
XSTC-22 (SPEX)	Mo, Cd

Supplementary Table 2. Items with correlations (*p* value < 0.05) in both genders for each group. Correlation analyses were performed separately for male and female, and items for which correlations were observed ($p < 0.05$) in both genders are shown.

Item	Element
Total-SeP (BD1)	Se
FL-SeP (AA3)	Se
Hemoglobin content	Fe
Mean corpuscular hemoglobin (MCH)	Fe
Mean corpuscular hemoglobin concentration (MCHC)	Fe
Urea nitrogen	As
Intake frequency: Bonito, Tuna	As
Food group: Bonito	As
Hemoglobin content	Mo
Hematocrit value	Mo
Food group: Whiskey	Mo
Intake frequency: Bonito, Tuna	Hg
Intake frequency: Breads	Hg
Food group: Seafood	Hg
Nutrient intake: Vitamin D	Hg
Food group: Bonito	Hg
Food group: Bread	Hg
Food group: Seafood	Hg

Item	Element
Total-SeP (BD1)	Se, Fe
FL-SeP (AA3)	Se, Fe, Hg
Hemoglobin content	Fe
Hematocrit value	Fe
Intake portion size: chocolate	Fe
Uric acid	As
Intake frequency: Horse mackerel, sardines	As
Food group: Seafood	As
Nutrient intake: Vitamin D	As
Food group: Horse mackerel	As
Food group: Seafood	As
Intake frequency: Bonito, Tuna	Hg
Intake frequency: Grilled fish	Hg
Food group: Bonito	Hg
